# Progress in osteoarthritis research by the National Natural Science Foundation of China

**DOI:** 10.1038/s41413-022-00207-y

**Published:** 2022-05-24

**Authors:** Yusheng Li, Wenqing Xie, Wenfeng Xiao, Dou Dou

**Affiliations:** 1 Deparment of Orthopedics, Xiangya Hospital, Central South University, Changsha, 410008 Hunan China; 2 National Clinical Research Center for Geriatric Disorders, Xiangya Hospital, Central South University, Changsha, 410008 Hunan China; 3 Department of Health Sciences, National Natural Science Foundation of China, Beijing, 100085 China

**Keywords:** Endocrine system and metabolic diseases, Metabolic disorders

## Abstract

Osteoarthritis (OA) in China is gradually becoming an important scientific research area that has had a significant impact on research and development (R&D) activities in the OA field worldwide. This article summarizes the R&D progress related to OA in China in recent years. The National Natural Science Foundation of China (NSFC) is a national funding institution for basic research and plays a critical role in promoting and supporting Chinese scholars’ R&D activities. We collected and analyzed information on NSFC funding in the field of OA from 2010 to 2019, including the amount, the level and the program categories of the funded projects. The data fully demonstrate the important and positive role of the NSFC in supporting free exploration, cultivating research teams and young talent, and boosting OA R&D. In this article, we outline and discuss hot topics in focused areas, key advances in this field and the prospects for progress in OA research in China.

## Introduction

Osteoarthritis (OA), also known as degenerative joint disease, is a leading cause of pain and disability and a source of socioeconomic costs among individuals over 40 years old worldwide.^1^ At present, OA has been listed as the fastest-growing major health condition, and the World Health Organization (WHO) has classified it as the second leading cause of disability.^2^

## Epidemiology and burden of OA

The prevalence of OA in various studies differs depending on the defined type of OA (self-reported, radiographic and symptomatic) and the age range, sex ratio, location and country of the study group.^3^ Generally, radiographic OA usually has a higher prevalence than symptomatic OA. Older women are more prone to have OA than their male counterparts. In 2017, a study on the prevalence of OA in 195 countries and territories found that the age-standardized prevalence of OA in the USA was 6 128.1 cases per 100 000 population.^4^ The overall OA prevalence (any form of OA except OA of the spine) was 26.6% for the Skåne region in the southern part of Sweden in 2012.^5^ In another study in the Netherlands, the prevalence of OA was reported to be 7% in 2007 but may increase to 12% by 2040 based on the current trend.^6^

With the growth and aging of the world’s population, it is estimated that the prevalence of OA will increase from 26.6% to 29.5% in every type of joint (13.8% to 15.7% for the knee joint and 5.8% to 6.9% for the hip joint) among the middle-aged population by 2032.^5^ The knee joint is generally considered the site most affected by OA, followed by the hand and hip joints.^7^ Ten percent of men and 13% of women (age 60 and older) were diagnosed with knee joint OA in the USA in 2010.^8^

Most of the large population-based observational studies evaluating OA prevalence were from North America and Europe, but these seriously lacked input from the epidemiological data of less developed countries and regions.

Previous publications reported knee pain rates of 2% in Pakistan in 1997 and 15%–18% in Oman in 1992.^9,10^ Approximately 20% of the studied population in Iran in 2006 had OA in at least one joint.^11^ Clarifying the epidemiological characteristics of OA in China is indispensable in policy formulations for the prevention, diagnosis, treatment and management of the disease. Although human joints are different, with the acceleration of the aging process in China, the prevalence of OA shows a trend consistent with that of the aging population.^7^ Obviously, with the continuous extension of life expectancy, the prevalence of OA will increase significantly in the future.^12^ China has a vast territory and a large population, making it difficult to carry out large-scale, multicenter and epidemiological research on the prevalence of OA. Previous studies were limited to one city or province, and only knee joint OA was analyzed. According to a population-based meta-analysis conducted in 2018 (published in a Chinese journal), a report on the prevalence of knee joint OA summarized from existing articles showed that the prevalence of knee joint OA in China was 17% (12.3% in males and 22.2% in females).^13^ There was no significant difference in the prevalence between northern and southern China or between rural and urban China. In another recent study in 2019, which included a summary of the existing publications, the overall prevalence of lumbar joint OA ranked as the most prevalent OA type, with a prevalence of 25.03%, followed by knee joint OA (21.51%), cervical joint OA (20.46%) and hand joint OA (8.99%) in Chinese middle-aged and elderly populations.^14^

Several studies compared the prevalence of OA between China and the USA. According to the Beijing OA Study, using a population-based survey conducted at the beginning of this century in central Beijing, the prevalence of radiographic knee joint OA was 42.8% in women and 21.5% in men,^15^ radiographic hip joint OA was 0.9% in women and 1.1% in men,^16^ and radiographic hand joint OA was 47.0% in women and 44.5% in men.^17^ Older Chinese women had a higher prevalence of knee joint OA than women in Framingham, Massachusetts (the Framingham OA Study), while the prevalence in men was comparable.

The prevalence of hip and hand joint OA in older people in Beijing was lower than the prevalence of hip and hand joint OA in the white population in the USA (in Framingham, Massachusetts).^18^ Lin et al. found that the prevalence of symptomatic knee joint OA in Chinese rural areas (i.e., Wuchuan County, Inner Mongolia) was much higher than that reported in Chinese urban regions (Beijing OA Study) or the Framingham OA Study.^18^

Presently, despite pain management and surgical intervention for patients in the last stage, OA remains the most challenging joint disease without effective therapeutic strategies.^19^ The focus of therapeutic treatment in OA is to reduce pain and improve the function of the affected joints.^20^ In patients over 65 years of age, the risk of mobility disability caused by knee joint OA is greater than that of any other disease.^21^ The initial management of OA includes nonpharmaceutical treatments targeting weight loss, lifestyle changes, physical therapy and so on, followed by pharmaceutical treatments such as the use of simple analgesics: acetaminophen, nonsteroidal anti-inflammatory drugs (NSAIDs), corticosteroid injections, hyaluronic acid (HA) injections and glucosamine.^22^ However, these treatments are far from satisfactory, as they are not powerful enough to modify the course of the underlying disease and are unable to stop the process of cartilage degeneration or prevent hazardous side effects.^23^ Oral administration of NSAIDs carries a substantial risk of clinical AEs, such as renal toxicity and gastrointestinal effects (ranging from mild heartburn to serious obstruction, ulceration, perforation and bleeding).^24^ Oral tramadol was found to be associated with a significantly higher rate of mortality.^25^ Even intraarticular hyaluronate was not recommended for the treatment of knee joint OA in older adults.^26^ If routine treatment is ineffective and pain interferes with the patient’s daily life, joint replacement surgery is recommended. There is evidence that joint replacement is effective and cost-effective in the clinic; however, its functional and clinical outcomes might be poor.^27^

OA can cause pain and activity limitations and significantly reduce quality of life, imposing a great burden on individuals and their family members. The side effects of OA can lead to participation restrictions, negative mood, fatigue, insomnia, and other problems.^28–30^ According to the 2017 Global Burden of Disease (GBD) Study, OA accounts for approximately 1.0% of all disability-adjusted life-years (DALYs) and 6.9% of all DALYs caused by musculoskeletal conditions.^31^ Nearly 80% of OA patients have movement limitations to some extent, and 25% of them are unable to accomplish daily activities. Knee joint OA accounts for 83% of the total burden caused by OA,^32^ and 11%–14% of these individuals require assistants to attend to personal care and routine needs.^33^ According to a recent study, people with either symptomatic or radiographic knee joint OA were at an increased risk of all-cause mortality.^34^

It was estimated that OA would cause an economic burden of 1% to 2.5% of the gross national product in North America, Europe and Australia.^35^ Kotlarz estimated that $185.5 billion per year would be used for medical care by patients with OA.^36^ However, the actual burden of OA is often severely underestimated. While we were amazed at this great financial loss caused by OA, we merely saw the tip of an iceberg. The indirect cost might be more than eight times the direct cost.^37^ The economic burden analysis of lower extremity OA worldwide shows that the weighted average annual total cost, direct cost and indirect cost for each patient are 85 000 euros, 67 000 euros and 54 000 euros/year, respectively.^38^ In the USA, the annual number of subjects who undergo the total knee replacement procedure is expected to increase from more than 1 million (approximate cost of $15 billion) in 2012 to more than 3 million by 2030.^39^ Considering that people undergoing arthroplasty are becoming much younger and that revision surgery is becoming more common at the same time, this cost will undoubtedly be increased.^39^ The revision procedures require longer operation times, more expensive prostheses, and longer patient stays in the hospital; they also pose greater risks of complications and morbidity. All these factors result in higher expenses and an increased burden on society. In Hong Kong, China, OA leads to a direct economic burden of HK $11 690–40 180 per person per year and an indirect economic burden of HK $3 300–6 640 per person per year.^40^ In the Chinese population over 60 years old, the loss of life expectancy caused by disability caused by OA is 0.27 years for men and 0.48 years for women.^41^ Data from representative national studies in China also show the high burden of OA (knee) in the Chinese population.^42^ The latest study on the change in disease burden of OA in China shows that the prevalence rate, the number of patients and the age-standardized years lived with disability (YLD) rate of OA have increased significantly over time.^43^ Specifically, the number of patients increased from 26.1 million in 1990 to 61.2 million in 2017, greatly exceeding the increase in the total population. The age-standardized prevalence rate increased from 2.9% in 1990 to 3.1% in 2017. The age-standardized YLD rate of OA per 100 000 people increased from 92.5 in 1990 to 98.8 in 2017.^43^

According to a study conducted in the USA in 2012, the mean episode-of-care payments ranged from US $25 568 for primary total joint replacement in patients without comorbidities to US $50 648 for revisions in patients with severe comorbidities or complications.^44^ It has been estimated that in the US, by 2030, the demand for primary TKA procedures will increase by 673% and that for revision surgeries by 601%, while the demand for primary THA procedures will increase by 174% and that for revision surgeries by 137% compared with the amount in 2005.^39^ By 2030, hospital costs for TKA revisions in the US Medicare population will exceed US $2 billion.^45^ In the United Kingdom, the number of primary and revision TKA surgeries has been predicted to increase by 117% and 332%, respectively, while increases of 134% and 331% in primary and revision THA surgeries were respectively predicted for the period between 2012 and 2030.^46^ It was predicted that the incidence of THA will increase from 332 per 100 000 people to 784 per 100 000 people by 2030 in Sweden.^47^ Although there is no national artificial joint registration system in China, the number of artificial hip and knee replacement operations in China exceeded 900 000 in 2019 and is still growing rapidly at a rate of nearly 20% per year, according to the incomplete statistics of Beijing Union Medical College Hospital.^48^

OA is becoming a growing source of workforce absenteeism, during which the cost is estimated to be as high as $10 billion in the US annually.^49^ In Australia, in 2009, approximately 80 000 workers retired from the labor force prematurely due to arthritis (and OA), which caused losses of AU $3.8 billion in private income, AU $291 million in social security payments and AU $394 million in personal income tax for the government.^50^ The impact of early retirement will cause great sociometric losses to the entire nation and the whole world. According to census data from 2019, among China’s 1.4 billion people, the population over 50 years of age accounts for 33.4%, and the population over 65 years of age accounts for 12.6% of the total.^51^ Considering the high prevalence of OA among elderly people in China and the rapid progress of population aging, it is expected that OA will become a heavy social and economic burden in China in the near future.

## The role of NSFC in promoting the research development in the field of OA in China

In 1986, at the beginning of China’s economic reform, the NSFC was set up to promote Chinese research activities in the basic science field. In addition, to respond to the call to improve public health nationwide and accelerate biomedical and translational research, the NSFC launched the Department of Health Sciences in 2009 to support basic and transformed medical and scientific research related to disease prevention, control and treatment in China. Since then, the NSFC has consisted of eight departments, namely, the Department of Mathematical and Physical Sciences, Department of Chemical Sciences, Department of Life Sciences, Department of Earth Sciences, Department of Engineering and Materials Sciences, Department of Information Sciences, Department of Management Sciences and Department of Health Sciences.

The funding portfolio of the NSFC includes four categories of programs, namely, exploration, talent, instrument and convergence. They have their own preferential focuses and together constitute the NSFC’s integrated funding system. The Exploration Programs include the General Program, Key Program, Major Program and Major Research Plan. The purpose of the Exploration Programs is to realize the innovative achievements of basic research, foster the balanced and coordinated development of disciplines, promote interdisciplinary research and stimulate original innovation. The Talent category supports young researchers in conducting independent research and assists researchers in regions with weak basic research. Its purpose is to cultivate an excellent academic backbone, top talent and innovative research teams to enhance China’s future scientific and technological competitiveness. The Instrument Program aims to strengthen research facilities, in particular, to support the development of indigenous scientific instruments, expand research areas and promote source research. The convergence programs are mainly oriented to meet scientific frontier and national needs, focus on major issues in basic research, promote disciplinary intersection and integration, integrate limited resources, gather and train high-level talent, create a scientific research highland while guiding social resources, solve common problems in basic research, and promote independent innovation capabilities in relevant fields, industries, or regions.

NSFC funding has promoted the progress of OA programs in China. The information contained in this article is helpful for the public and research communities to understand the current and future research trends and can help policy-makers formulate new funding policies to promote the further development of OA programs worldwide.

### Launching continuous funding support in the field of OA

**Fig. 1:**
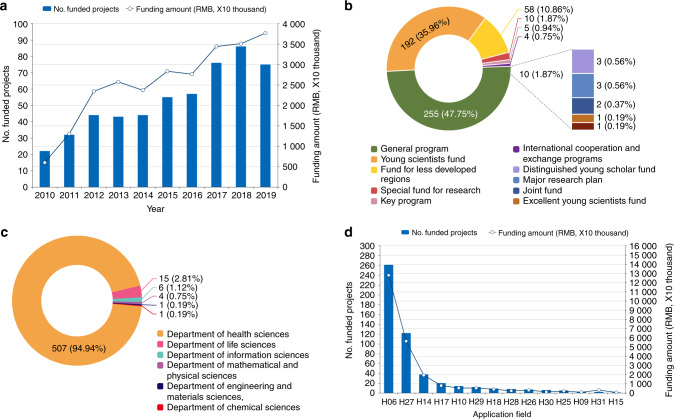
Statistics for the situation of funding in the field of OA provided by the NSFC in China (2010-2019). **a** The annual number of funded projects and their gross funding amounts. **b** The number of funded projects related to OA by the different project types. **c** Comparison of different departments of the NSFC involved in OA research. **d** The rank of codes initiating grants for the study of OA

In the past decade (2010–2019), the NSFC initiated 534 various research projects with a total funding of 254.85 million Ren Min Bi (RMB) (approximately US $36.41 million) to support exploration in the OA field. Since 2015, the funds for projects have been divided into direct costs and indirect costs. Only the direct costs were allocated to the principal investigators so that the funding data from 2015 to 2019 included purely direct costs. Figure 1a demonstrates the development track of the annual number of funded projects and their gross funding amount. The number of funded projects experienced a healthy year-to-year increase. In 2010, the number of funded projects was 22, and the number almost quadrupled to 86 in 2018. We also noticed that the funded grants in 2012, 2013 and 2014 remained at the same level. This situation was mainly attributed to the new rule formulated in 2013, which stipulated that if the general project proposal has not been funded for two consecutive years, the investigators have no choice but to wait for one year before resubmitting a new proposal.

The annual funding amount also increases with the number of research grants, particularly after 2012 (Fig. 1a). From 2010 to 2019, the government’s funding through the NSFC increased at an alarming rate of 22% every year. Considering the average funding standard of the projects (taking the general program as an example), the budget for each general program before 2011 was only 300 000 RMB (US $42 900), and after 2011, it was elevated to 800 000 RMB (US $114 300). The data show that the funding level of individual projects in China is equivalent to that of some developed countries.

Forty-eight percent (255 projects) of OA-related funded projects belong to the “General Programs” funding scheme, which is the main funding type of NSFC, allowing funded scientists to freely choose their research topics (Fig. 1b). The Young Scientists Fund scheme (36%, 192 projects) is the second-largest project type, enabling young scientists to freely research key scientific fields. In addition, 58 research projects belong to the Fund for Less Developed Regions, which support scientists from the provinces of Inner Mongolia, Ningxia, Xinjiang, Tibet, Qinghai and Gansu. These data indicate that the NSFC has developed corresponding funding strategies for scientists at different stages of development as well as for different regions.

Interestingly, six departments of the NSFC were involved in OA research (Fig. 1c), which indicated that OA is a multidisciplinary and widely studied field with a high possibility of cross-integration. The Department of Health Sciences plays a leading role in OA research, with 507 (95%) funded projects. Before 2021, there were 31 primary application codes (H01 to H31) in the Department of Health Sciences, covering all organ systems and various medical categories (details in Table S1). Funded grants in OA-related studies are distributed in 14 primary application codes (Fig. 1d). The main application code, H06, which stands for “Abnormalities and Diseases of Locomotor System”, undoubtedly took first place in the ranking, with 261 projects and 127.88 million RMB funding. This was followed by code H27, with 122 projects belonging to traditional Chinese medicine.

**Fig. 2:**
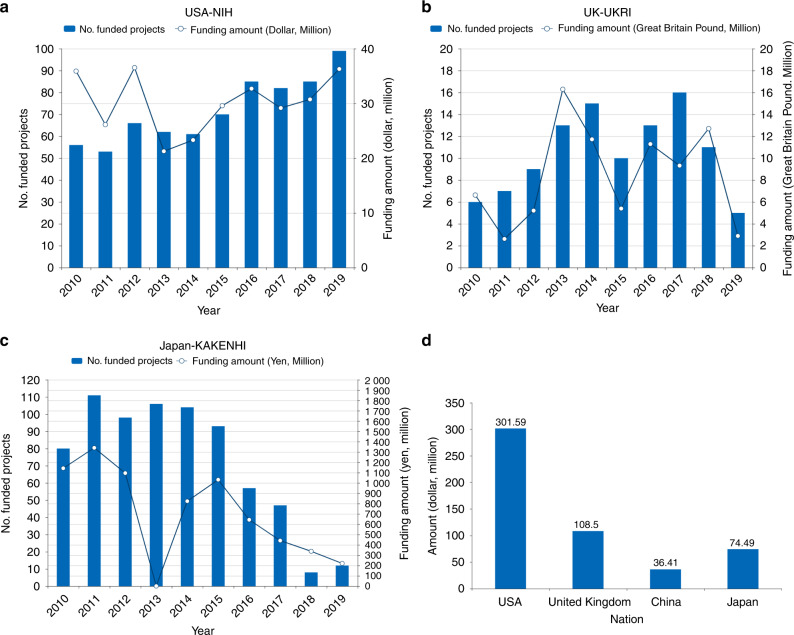
Comparison of national basic research grant support in the field of OA among the NIH, UKRI and KAKENHI (2010–2019). **a** Changes in the number of funded projects and funding amounts of the NIH in the USA. **b** Changes in the number of funded projects and funding amounts of UKRI in the United Kingdom. **c** Changes in the number of funded projects and funding amounts of KAKENHI in Japan. **d** Comparison of the amounts of grants allotted for OA research in the USA, United Kingdom, China and Japan

In comparison, between 2010 and 2019, the National Institutes of Health (NIH) of the USA funded 719 OA-related projects, with a total amount of US $301.59 million (Fig. 2a, d). In the United Kingdom, UK Research and Innovation (UKRI) has allocated 84 million British pounds (US $108.50 million) for 105 OA research projects (Fig. 2b, d). Government funding support for OA research in Japan from the national Grants-in-Aid for Scientific Research (KAKENHI) trailed behind that of the NIH and UKRI with 8.17 billion yen (US $74 million) (Fig. 2c, d).

### Supporting research scientists nationwide

**Fig. 3:**
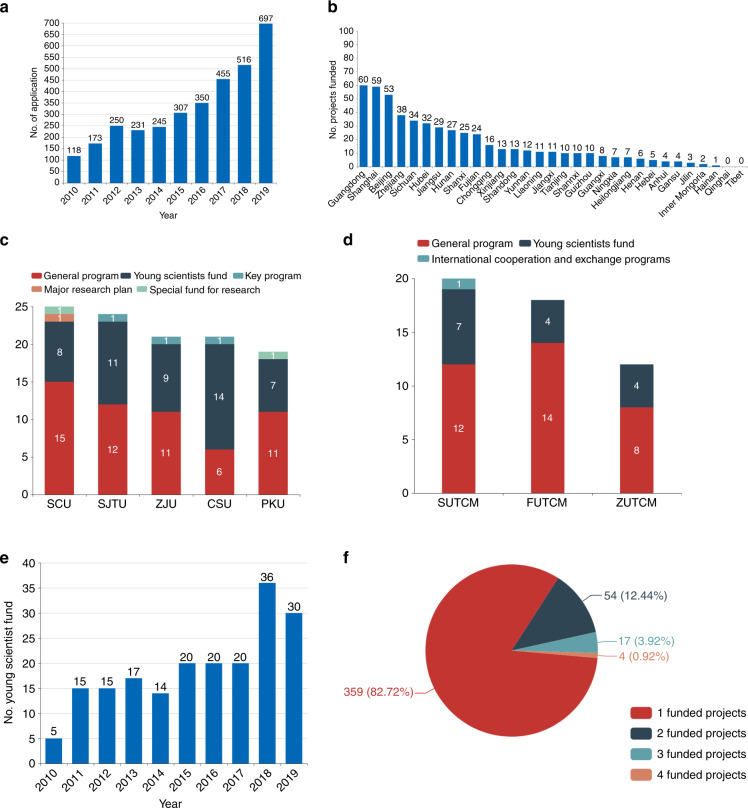
Statistics for the number of funded projects in different regions and universities in China (2010–2019). **a** The project applications are related to OA. **b** The comparison of different provinces of mainland China with respect to the number of obtained projects. **c** The top five universities that received osteoarthritis-related funds from the NSFC. **d** The top three traditional Chinese medicine universities by type of funded projects. **e** The changing trend in the number of young scientist projects. **f** Statistics for the number of scholars who received financial support in the field of OA

The NSFC encourages every researcher to submit his or her own scientific proposal. However, few scientists in the field submitted project applications in the first year of the decade. With the continuous implementation of this policy, an increasing number of scientists and orthopedic surgeons have submitted their own research proposals. Project applications related to OA in 2019 displayed a rapid expansion to six times that in 2010 (Fig. 3a). The affiliations of the applicants were another remarkable change. In 2010, only 17 colleges or research institutes had researchers who submitted scientific proposals, while in the past few years, the number of colleges or research institutes has increased to 64, and a total of 697 proposals related to OA research were submitted.

Over the past 10 years, the NSFC has funded 129 host institutions in the OA field. Except for Qinghai and Tibet, the provinces of mainland China all obtained national support sources. Most of these institutions are centralized in developed areas of China. Among the OA projects funded by the NSFC, economically developed provinces and cities (including Guangdong, Shanghai and Beijing) account for more than 32.2% of the total funded grants. In contrast, fewer than five projects in relatively underdeveloped provinces (such as Inner Mongolia and Hainan) have been funded (Fig. 3b).

The top research universities designated in the national “985” and “211” strategic development plans for tertiary education account for a high proportion of funded projects. For example, the top five universities that received OA-related funds from the NSFC are Sichuan University (SCU, 25 projects, Sichuan), Shanghai Jiao Tong University (SJTU, 24 projects, Shanghai), Zhejiang University (ZJU, 21 projects, Zhejiang), Central South University (CSU, 21 projects, Hunan) and Peking University (PKU, 19 projects, Beijing) (Fig. 3c). With the existing research infrastructures and the ability to compete for centralized funding, these universities have great strength in OA research in China.

As mentioned above, the second-largest division to support OA study was traditional Chinese medicine. Traditional Chinese medicine is a unique resource in China. Other countries, such as Japan and the USA, have no or few funded projects in herbal medicine, which reflects a divergence of interest or strength. Among 23 universities of traditional Chinese medicine, 19 have already been funded projects related to OA. Each of the top three received more than 10 funded projects. They are Shanghai University of Traditional Chinese Medicine (SUTCM, 20 projects, Shanghai), Fujian University of Traditional Chinese Medicine (FUTCM, 18 projects, Fujian) and Zhejiang University of Traditional Chinese Medicine (ZUTCM, 12 projects, Zhejiang) (Fig. 3d).

### Cultivating an increasing number of research teams and young talent

Generally, the implementation of a project requires a principal investigator, 2~4 formal researchers and 1~4 graduate student participants. Therefore, the implementation of the project enables at least two researchers and one graduate student to participate and receive certain training. To date, 534 projects have been funded by the NSFC in the OA field, which would have helped cultivate approximately 2 000 researchers and 900 graduate students. We are pleased to find that several leading research teams have formed from the data of funded projects. For example, the OA research teams from Southern Medical University, Central South University, Fourth Military Medical University and Fujian University of Traditional Chinese Medicine have all won more than 10 projects in the OA field, showing the team’s strengths. The NSFC has played an important role in cultivating OA research teams in our country.

The NSFC attaches great importance to young scientists’ funds that help cultivate young talent. With the “first bucket of gold” in research, young generations can initiate their own research ideas and accumulate an academic foundation. At the early stages, there were few youth proposals, so there was little funding. In terms of number, only five young scientist projects were funded in 2010. After 2015, the number of funded projects increased rapidly, exceeding 20 per year (Fig. 3e). After their youth funding ends, young scientists may have a basis to apply for a general program. According to incomplete statistics, 15% of youth fund recipients in the OA field have again received a general program grant in the same discipline. Therefore, youth projects have developed into the most important talent pool and play an important role in promoting young researchers to become qualified scholars.

We are also pleased to find that some scholars have chosen to devote themselves to OA year-round and have received repeated financial support, which can also help them focus on this direction toward breakthroughs. In the field of OA, four researchers were funded four times, 17 scholars were funded three times, and 54 scholars were funded twice by the NSFC (Fig. 3f).

Moreover, there are two kinds of funds, especially for excellent young scientists and distinguished young scholars, based on the scouting out of these brilliant emerging researchers. These two programs support young scholars with good or outstanding achievements in basic research to conduct innovative investigations in areas of their own choice to promote the fast growth of creative young talents and foster a number of outstanding talents in international science frontiers. These researchers were selected mainly through three criteria: (1) Extraordinary creativity. (2) An excellent track record and a likelihood to make significant progress in the future. (3) Great interest in developing relevant research directions or branches of disciplines. The selection process is very rigorous, and the number of awardees is quite limited. As of December 31, 2019, three scholars in the OA field were granted funding from the National Science Fund for Distinguished Young Scholars, and one scholar was granted funding from the National Science Fund for Excellent Young Scholars. They have become academic leaders and top scientists in OA research in China.

## Advances in OA research in China

### Publication trends of OA research worldwide

**Fig. 4:**
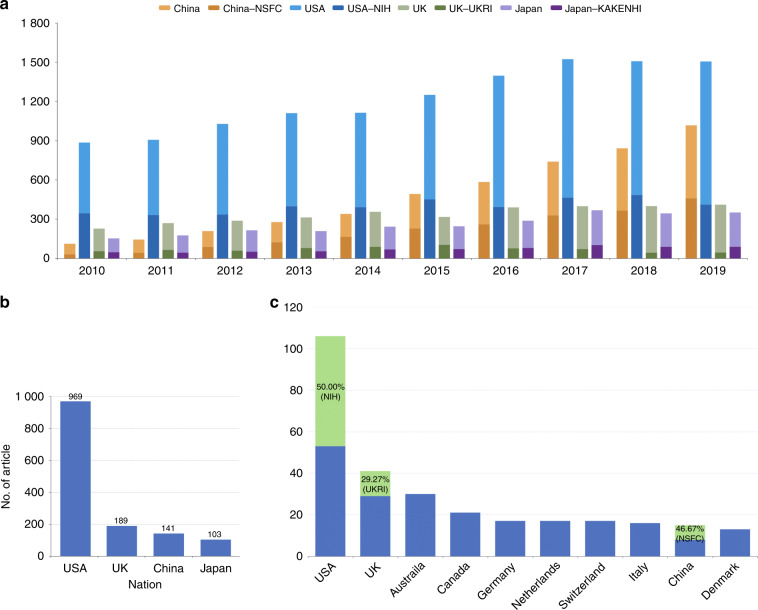
The publication situation of China and other countries in the field of OA (2010-2019). **a** The number of publications from China, the USA, the UK and Japan and the proportion funded by the NSFC, the NIH, UKRI and KAKENHI. **b** Comparison of the numbers of high-level publications among the top four countries. **c** The data of highly cited papers from different countries in the Web of Science

Funding began to pay dividends in terms of publications over the past decade. We searched the Web of Science (http://apps.webofknowledge.com/) for OA research publications from 2010 to 2019 and showed that an increasing number of articles have been published in the OA field internationally in the past 10 years. As shown in Fig. 4a, publications from China have increased almost 10-fold, a jump from 111 in 2010 to 1 017 in 2019. In 2010, there were 2 478 articles in this field worldwide, and China ranked eighth, accounting for 4.4%. Ten years later, while still behind the USA, China’s publication output (accounting for 19.1%) has already outpaced any other country, including England (410 publications) and Japan (351 publications) (Table 1).

**Table 1:** Top 10 nations for OA-related publications in 2010 and 2019

NO.	2010	2019
1	USA (885)	USA (1 519)
2	England (227)	Peoples R China (1 017)
3	Germany (216)	England (410)
4	Canada (182)	Japan (351)
5	Japan (152)	Germany (328)
6	Australia (145)	Australia (280)
7	Netherlands (122)	South Korea (278)
8	Peoples R China (111)	Canada (272)
9	Switzerland (102)	Italy (238)
10	France (100)	Netherlands (209)

As one of the chief funding sources for basic research in China, the NSFC has played a vital part during this rapid development period. From 2010 to 2019, a total of 2 084 papers were published with the support of at least one grant from the NSFC in the field of OA, accounting for 43.8% of those in China. The quantity of SCI papers supported by the NSFC increased by at least 15 times, from 29 in 2010 to 458 in 2019. Compared with the NIH in the USA and KAKENHI in Japan, the ratio of SCI publications in OA research supported by the NSFC was only 26.1% in 2010, while those of the NIH and KAKENHI were 39.0% and 30.2%, respectively. Interestingly, the ratio of NSFC-funded publications increased dramatically, reaching 45.3% in 2019. However, both the NIH and KAKENHI were reduced to below 30% (Fig. 4a).

To gain an appreciation for high-level publication trends in OA research, we also collected and analyzed published papers from top journals during 2010-2019. We defined the top 5-10 journals in each category related to OA basic research from the Journal Citation Reports database as “Top Journals”. For example, *Nature Medicine, Science Translational Medicine, Journal of Clinical Investigation, Trends in Molecular Medicine, Journal of Experimental Medicine, EMBO Molecular Medicine* and *Annual Review of Medicine* were regarded as the top journals in the category “Medicine, Research & Experimental”. In terms of category, “Rheumatology”, *Nature Reviews Rheumatology, Annals of the Rheumatic Diseases, Arthritis & Rheumatology, Rheumatology* and *Osteoarthritis and Cartilage* were selected as the top journals. A total of 31 journals from six OA-related categories were finally chosen as the top journals in basic OA research (Table S2). In recent decades, the USA published 969 articles in these OA top journals, far more than the UK, China and Japan (Fig. 4b). Similarly, from the data of highly cited papers in 2010-2019 on the Web of Science, the USA produced 106 articles from all 187 papers. The top 10 countries that published highly cited papers are shown in Fig. 4c, and China ranked ninth. The ratio of NSFC-funded highly cited papers among Chinese publications was 46.7%, similar to the NIH of the USA (50.0%) and far more than that of UKRI of the UK (29.2%) (Fig. 4c).

### Publication trends of high IF literature from China in elite journals

**Fig. 5:**
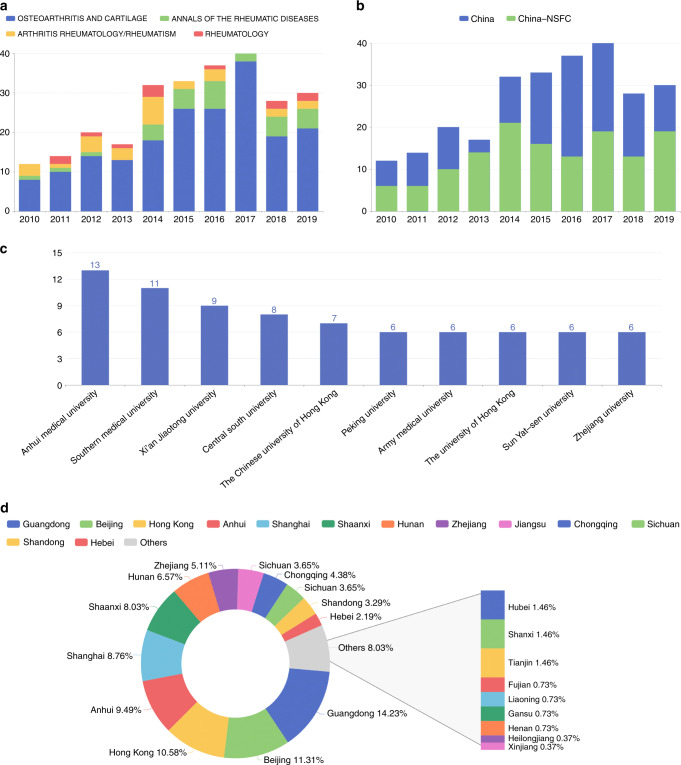
Statistics for publications about China in the field of OA (2010–2019). **a** Statistics on the number of papers published in four elite journals of rheumatology. **b** Number of Chinese studies published in elite rheumatology journals and the proportion of NSFC funding. **c** Statistics on the affiliation of the corresponding authors of articles in elite rheumatology journals. **d** Distribution of the corresponding authors’ affiliations in China by province

In the last decade, the contribution of Chinese scholars to OA research in the top journals of rheumatology (i.e., *Annals of the Rheumatic Diseases, Arthritis & Rheumatology* (formerly *Arthritis & Rheumatism)*, *Rheumatology* and *Osteoarthritis & Cartilage*) has increased significantly. In 2010, there were only 12 papers from China in these four elite journals. This beginning is in sharp contrast to the situation in 2017 (40 Chinese papers were published in 2017) (Fig. 5a). In the past few years, Chinese researchers have often published papers in the top journals in the field of OA. For example, in 2019, Chinese authors published 5, 2, 2 and 21 papers in *Annals of the Rheumatic Diseases, Arthritis & Rheumatology, Rheumatology* and *Osteoarthritis & Cartilage*, respectively (Fig. 5a). Most of China’s influential papers on OA were published in *Osteoarthritis & Cartilage* (193 out of 263, 73.3%), the official journal of Osteoarthritis Research Society International (Fig. 5a). It is noteworthy that among the 263 papers published in these four journals from China, 137 (52.0%) were supported by the NSFC, indicating the significant role of the NSFC in accelerating and supporting OA research in China (Fig. 5b). Based on the further analysis of these 263 papers, we found that the first author/corresponding author (including co-first/co-corresponding author) in 137 papers belonged to Chinese institutions, and then we refined this analysis according to the standards (see Table S3 for details). Anhui Medical University, Southern Medical University, Xi’an Jiaotong University and Central South University are the research institutions with the most publications published in those four elite journals, with 13, 11, 9 and 8 papers published, respectively (Fig. 5c). Guangdong, Beijing, Hong Kong, Anhui and Shanghai not only are the provinces that have become gathering places for medical research institutions but also account for 54.38% of the Chinese papers published in these top journals (Fig. 5d), which have made great contributions to promote the development and progress of OA research in China.

### Representative achievements of OA research in China

Between 2010 and 2019, the NSFC provided a major boost to research in the field of OA in China. Here, we summarize the representative achievements of NSFC awardees in this decade in the several aspects.

OA is one of the most common health problems in older adults worldwide. However, the current research on OA is not comprehensive enough. Epidemiological studies and experiments not only may yield new research directions but also can help us update the guideline information for OA. Epidemiology will provide more accurate guidance to the clinical management of OA. Here, we summarized the representative epidemiological studies performed by NSFC funding recipients over the last decade. A cohort analysis study of patient data collected by Lei G et al. showed that compared to other commonly used nonsteroidal anti-inflammatory drugs, tramadol might increase all-cause mortality in patients with OA. This research had a significant impact on domestic and international guidelines for the treatment of OA.^25^ Ding C et al. showed that vitamin D supplementation has no obvious effect on the loss of tibial cartilage in patients with knee OA, causing people to re-examine the role of vitamin D.^52^ In addition, Zhang H et al. found a significant correlation between IL-7 and OA in a retrospective case–control analysis of a Chinese Han population.^53^

Previously, there was a large gap in the understanding of the pathological mechanism of OA. This large gap affected the accuracy of the diagnosis and treatment of OA and greatly affected the patients’ quality of life. Currently, the pathogenesis of OA has been exhaustively studied by NSFC awardees and has brought new insights to the field. Bai X et al. investigated the effects of the mTORC1 signaling pathway and the Fyn-activated β-catenin pathway on OA.^54–56^ Another study showed that synovial macrophage M1 polarization exacerbates experimental CIOA partially through Rspo2.^57^ Chen L et al. demonstrated a unique role of fibroblast factor receptor 3 and fibroblast factor receptor 1 in the regulation of chondrocytes in OA.^58,59^ Ouyang H et al. revealed the regulatory role of chitosan microspheres and OCRL1 on Rac1 in OA.^60,61^ In addition, they focused on the field of epigenetics and found an important role of kdm6b in OA.^62^ Jiang Q et al. revealed that the inhibition of PPARγ plays a key role in the development of OA and that PPARγ preservation has therapeutic potential for OA.^63^ Su P et al. studied a novel mechanism, finding that COL2A1 inhibits chondrocyte hypertrophy and suggesting that a decrease in COL2A1 may initiate and promote the progression of OA.^64^ NSFC awardees also identified a reciprocal antagonism between the Hippo-YAP/TAZ and NF-κB signaling pathways in regulating the induction of matrix-degrading enzyme expression and cartilage degradation during OA pathogenesis.^65^ The study by Wang Y et al. was the first to perform high-precision single-cell transcriptome sequencing in cells from different pathological sites in patients with OA.

Moreover, they determined the importance of HPIP for the development of OA.^66,67^ Xie J and his colleagues explained the contribution of the meniscus to the progression of OA through changes in the meniscus in a mouse model induced by anterior cruciate ligament transection (ACLT) and provided clues for the early initiation of preventive treatment for OA.^68^ Fang S et al. demonstrated that overexpression of CircSERPINE2 alleviated the apoptosis of HCs via miR-1271-E, providing a potentially effective therapeutic strategy.^69^

At present, routine orthopedic treatment still needs to be improved. Many patients are inevitably headed toward the outcome of surgery. Fortunately, NSFC awardees have made great strides in diagnosing and treating OA in recent years. Xing L et al. showed that the use of longitudinal ultrasound to quantify posttraumatic OA has great clinical significance and potential. These studies will greatly enrich the diagnostic and therapeutic evaluation of OA.^70^ Ao Y et al. discovered that BNTA (a small molecule with ECM modulating function) could induce SOD3 expression to inhibit OA development, showing its therapeutic promise. In addition, in the field of epigenetics, they discovered the potential of lncRNA-CIR, lncRNA-MSR and miR-101 as therapeutic targets for OA.^71–74^ Chang J et al. found that the biokinetics of silicates could stimulate angiogenesis and ossification. Subsequently, they designed 3D-printed Sr_5_(PO_4_)_2_SiO_4_ (SPS) bioactive ceramic scaffolds, which are important for cartilage and subchondral bone reconstruction.^75,76^ Ouyang H et al. developed a lithium-containing mesoporous bioglass (Li-MBG) scaffold that facilitated the regeneration of osteochondral defects and discovered that a radially oriented collagen scaffold with SDF-1 promotes osteochondral repair by facilitating cell homing.^77,78^ Yang L et al. revealed that miR-100-5p-abundant exosomes derived from infrapatellar fat pad MSCs could maintain cartilage homeostasis and protect articular cartilage from damage by regulating the mTOR-dependent autophagic pathway.^79^ Zhou C et al. found that Runt-related transcription factor-1 (Runx1) may be an effective therapeutic target for OA and osteoporosis, when exploring the role of Runx1 in osteoarthritis induced by ACLT surgery.^80^

### Co-occurrence analysis of OA study

**Fig. 6:**
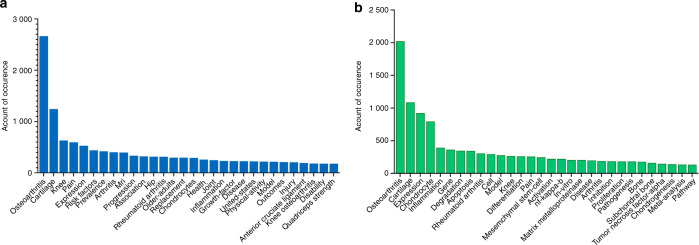
The distribution of the top 30 keywords in the publications supported by the NIH or NSFC. **a** The distribution of the top 30 keywords in the publications supported by the NIH. **b** The distribution of the top 30 keywords in the publications supported by the NSFC

Co-occurrence analysis indicates that the relationship of items is built based upon the number of publications in which they occur together. The purpose of co-occurrence analysis is to discover directions and popular topics in research and has proven to be important for monitoring the development of science and programs. Keywords (defined as words that were used more than five times in titles and abstracts in all publications) used in all included studies were analyzed using VOSviewer. As shown in Fig. S1a, the 1 000 identified keywords from the NIH studies were classified into four clusters: first, the orange clusters focused on pathway and mechanism studies; second, the green clusters were mainly clinically relevant pathological features and prognostic analysis; next, the purple clusters concerned rehabilitation and sports medicine-related content; and finally, the yellow clusters were mostly related to imaging content. As shown in Fig. S1b, of the 618 identified keywords in the studies, we know that the NSFC-funded study focused more on basic research, such as pathology and pathogenesis, OA, cartilage, expression, chondrocytes and inflammation, occupying the top five keywords. This result also coincides with the reality that the NSFC mainly supports basic research. Clearly, in OA, pathway and mechanism research, clinicopathological characteristics and prognostic analysis are the most common research areas funded by the NSFC or NIH. According to the distribution of keywords, NIH-funded studies were more focused on pathogenesis, risk factors, rehabilitation and radiology, while NSFC-funded studies concentrated more on the mechanisms of inflammation and cartilage degradation. The top 30 keywords related to basic research, such as MSCs, inflammatory factors and apoptosis, accounted for 23.56% of NSFC studies, while they accounted for 9.97% of NIH studies (Fig. 6a, b). This result may show us that while the NIH supports not only basic and translational research but also clinical research, the NSFC mainly supports basic and translational research.

## Future perspectives

The OA field has become an applied science that integrates multidisciplinary knowledge with rich connotations. Since China implemented its policy of reform and opening-up in the 1970s, the country’s economy has grown at an average rate of 9% in the past 30 years, which has tremendously promoted progress in the OA discipline. Economic development has become an important impetus to the advances in OA research. The NSFC already has six departments involved in OA research, which suggests that it is a multidisciplinary field. With the Department of Health Sciences playing a leading role in OA research, 95% of funded programs have covered all organ systems and various medical categories, including 14 codes of findings from OA research. The potential of changing clinical treatments by facilitating the development and testing of new strategies promotes the prevention and treatment of OA through multidisciplinary system-based approaches, including biology, mechanics and architecture.^81^ Tissue engineering can facilitate preclinical and clinical trials of OA through pathological tissue and animal model studies, which reflects a multidisciplinary fusion and a driving role in OA studies.^82^ Epidemiological and experimental studies have identified obesity as an important risk factor for OA, and studies of obesity-related metabolic syndrome further revealed the pathogenesis of OA, showing that multidisciplinary cross-fusion can greatly promote the depth and breadth of research on OA.^83^ Combining nanotechnology with OA has also greatly facilitated advances in OA diagnosis and articular cartilage imaging.^84^ Generous funding is a key factor and an important resource for more comprehensive or complex studies. In the future, the NSFC may further play a more important role in the development of the OA research field by supporting the development, testing, promotion and application of innovative technologies and drugs.

We have analyzed the important results of NSFC investment in the study of OA over the past few decades, which has contributing to future trends in NSFC funding for such research. Studies show that a high proportion of basic OA projects provide a rich knowledge base that is mature enough to be translated into clinical trials. OA clinical trials have been initiated by several researchers funded by the NSFC. However, the total number of clinical trials related to OA is still relatively small, reflecting that clinical trials are an emerging field of OA research with barriers to translating basic research into clinical practice information. Therefore, translating the findings of basic scientific research into clinical research is an important funding direction for the NSFC in the future. The prevalence of OA is rising, with constantly increasing risk factors. OA-associated joint pain leads to limited function, poor sleep, fatigue, low mood and so on.^85^ Therefore, the development of more effective and safer treatments is greatly needed for clinical research in OA. Joint replacement is an effective treatment for end-stage OA, although poor results and a limited lifespan of prostheses have been reported. Therefore, the focus has shifted to disease prevention and the treatment of early-stage OA. This task is challenging because traditional imaging techniques can detect only very advanced disease, and the relationship between pain and structural degradation is not close. The progress and development of imaging and biochemical markers of OA may provide the potential for developing new diagnostic and therapeutic measures, which provides the possibility for joint-protection interventions such as drugs and surgical methods.^1^

For example, theranostic nanosomes, nanoparticles and superparamagnetic iron oxide nanoparticle tracking may be an attractive treatment in the future.^86^ In vitro, shock wave therapy has shown efficacy in OA in animal experiments and clinical studies,^87^ and future applications in the field of OA have important clinical value. Tetrahedral framework nuclear acid/wogonin complexes can be used as an injectable formulation for the treatment of OA.^88^ After the intraarticular injection of brush-like cartilage-binding nanofibers with a hyaluronan backbone and two key side chains (lubricin and lipid), superior lubrication performance can promote cartilage regeneration and even reverse the development of OA.^89^ With the development and maturation of gene therapy technology, a DNA-grafted hyaluronic acid system with enhanced injectability and biological stability has also been designed and developed to treat photocontrolled OA.^90^ In the future, these applications in the field of OA will have important clinical value. With the development of biomedical technologies and the integration of multiple disciplines, it is possible to apply various new biomedical technologies in the OA field. The NSFC has been committed to supporting the development of interdisciplinary scientists in the field of OA.

With the innovation of technology, new technologies continue to emerge, providing new possibilities for OA research. The NSFC provides tremendous support and impetus for OA research in China by focusing on cutting-edge disciplines and technology development. Through high-precision single-cell transcriptome sequencing of cells from different disease sites of patients with OA, seven chondrocyte subtypes were discovered in human cartilage, including three cell subtypes with different functions and their molecular markers, which revealed the spatial distribution of these cell subtypes in cartilage tissue and their temporal distribution in the process of OA.^67^ Moreover, from the perspective of big data, using unsupervised cluster analysis, a research team found that the cartilage transcriptome of patients with OA can divide OA into four subtypes: the glycosaminoglycan metabolic disorder subtype (C1), collagen metabolic disorder subtype (C2), activated sensory neuron subtype (C3) and inflammation subtype (C4).^91^ This classification has a good correlation with clinical symptoms and imaging evidence. More importantly, OA therapeutic drugs can be well matched with the four subtypes of OA.^91^ In recent years, continuous progress has been made in the clinical transformation of stem cell therapy for OA. China has a few new stem cell drugs for OA clinical trial applications for approval. The use of stem cells for OA treatment in China is rapidly undergoing clinical transformation. Internationally, the clinical application of stem cell treatment for OA is also promising.^92^

Dysregulation of the gut microbiome promotes systemic inflammation. Recent studies have demonstrated an association between the gut microbiome and OA. An animal experiment investigated the gut-joint axis in OA, which may contribute to potential transformation opportunities by elucidating the role of the microbiome in the development of OA.^93,94^ Studies have also demonstrated gut microbiome biomarkers for identifying overweight populations at risk for OA.^95^ Some particular microbes (*Fusobacterium*, *Faecalibacterium* and *Ruminococcaceae*) have also been shown to play an important role in exacerbating OA.^96^ Robot surgical systems have a wide range of uses and many applications in clinical surgery. Surgeons can operate machines while stationed away from the operating table, and this approach will have an important role in future OA-related treatment, such as mechanical arm-assisted single-chamber knee replacement (UKA) and robot-assisted total knee replacement.^97,98^ Creative ideas will continue to provide the breadth and depth necessary for research on OA to further understand this disease.

With the increase in aging populations, OA, the most common type of arthritis, leads to the loss of human resources and places a heavy economic burden on families and society. It is particularly important to develop strategies for OA by deeply elucidating the pathogenesis of OA, finding effective targets for treatment, and intervening in the disease early. Since the establishment of the Department of Health Sciences in NSFC, the number and scale of researchers in the OA research field have increased significantly, contributing to the high level of research achievements. However, we should be aware that there is still a gap between basic research in the OA field in China and that in developed countries, and there are still many challenges and unresolved problems in OA research. With the support of the NSFC, the Chinese scientific community will make further contributions to research in the OA field by continuing to increase original, innovative exploration and cultivate an increasing number of research teams and young talents in the future.

## Supplementary information

Supplemental table 1

Supplemental table 2

Supplemental table 3

Figure 1

## Data Availability

All data generated for this study are available from the corresponding authors upon reasonable request.
