# Supplementary material for: Progress in osteoarthritis research by the National Natural Science Foundation of China
Source: Bone Res. 2022 May 24;10:41. doi: 10.1038/s41413-022-00207-y (PMC9130253; doi:10.1038/s41413-022-00207-y)
Supplement: Supplementary file 1 — Supplemental table 1 [file 41413_2022_207_MOESM1_ESM.docx]

Supplemental table 1. 31 primary application codes in the Department of Health Sciences (H01 to H31)

| Application Code | Field |
| --- | --- |
| H01 | Respiratory system |
| H02 | Circulatory system |
| H03 | Digestive system |
| H04 | Reproductive system/ perinatology/neonatology |
| H05 | Urinary system |
| H06 | Orthopedics and sports medicine |
| H07 | Endocrine system/metabolism and nutrition support |
| H08 | Blood system |
| H09 | Neurological and psychiatric diseases |
| H10 | Medical immunology |
| H11 | Skin and appendages |
| H12 | Ophthalmology |
| H13 | Otorhinolaryngology head and neck science |
| H14 | Oral and craniomaxillo-facial science |
| H15 | Emergency and intensive care medicine/trauma/ burns/plastic surgery |
| H16 | Oncology |
| H17 | Rehabilitation medicine |
| H18 | Medical imaging and biomedical engineering |
| H19 | Medical pathogenic microorganisms and infection |
| H20 | Laboratory medicine |
| H21 | Special medicine |
| H22 | Radiology |
| H23 | Forensic sciences |
| H24 | Endemiology/occupational medicine |
| H25 | Gerontology |
| H26 | Preventive medicine |
| H27 | Chinese medicine |
| H28 | Chinese materia medica |
| H29 | Integrated Chinese and western medicine |
| H30 | Materia medica |
| H31 | Pharmacology |
