# Supplementary material for: Progress in osteoarthritis research by the National Natural Science Foundation of China
Source: Bone Res. 2022 May 24;10:41. doi: 10.1038/s41413-022-00207-y (PMC9130253; doi:10.1038/s41413-022-00207-y)
Supplement: Supplementary file 2 — Supplemental table 2 [file 41413_2022_207_MOESM2_ESM.docx]

Supplemental table 2. Top journals for OA basic research

| Categories | Journals |
| --- | --- |
| Medicine, Research & Experimental | Nature Medicine |
|  | Science Translational Medicine |
|  | Journal of Clinical Investigation |
|  | Trends in Molecular Medicine |
|  | Journal of Experimental Medicine |
|  | EMBO Molecular Medicine |
|  | Annual Review of Medicine |
| Multidisciplinary Sciences | Nature |
|  | Science |
|  | National Science Review |
|  | Science Advances |
|  | Nature Communications |
|  | Nature Human Behaviour |
|  | Proceedings of the National Academy of the Sciences of the United States of America |
| Orthopedics | American Journal of Sports Medicine |
|  | Journal of Physiotherapy |
|  | Osteoarthritis and Cartilage |
|  | Journal of Bone and Joint Surgery-American Volume |
|  | Arthroscopy: the Journal of Arthroscopic and Related Surgery |
| Rheumatology | Nature Reviews Rheumatology |
|  | Annals of the Rheumatic Diseases |
|  | Arthritis & Rheumatology |
|  | Rheumatology |
|  | Seminars in Arthritis and Rheumatism |
| Sport Sciences | British Journal of Sports Medicine |
|  | Sports Medicine |
|  | Exercise immunology review |
|  | American Journal of Sports Medicine |
|  | Exercise and Sport Sciences Reviews |
| Cell and Tissue Engineering | Cell Stem Cell |
|  | Bone Research |
