# Supplementary material for: Progress in osteoarthritis research by the National Natural Science Foundation of China
Source: Bone Res. 2022 May 24;10:41. doi: 10.1038/s41413-022-00207-y (PMC9130253; doi:10.1038/s41413-022-00207-y)
Supplement: Supplementary file 3 — Supplemental table 3 [file 41413_2022_207_MOESM3_ESM.docx]

Supplemental table 3. Top journal articles on rheumatology published by Chinese scholars (first author/corresponding author (including co-first/co-corresponding author) )

| High-impact Rheumatology Journals | Publication Year | Article Title | Affiliation |
| --- | --- | --- | --- |
| Osteoarthritis & Cartilage | 2010 | The support of matrix accumulation and the promotion of sheep articular cartilage defects repair in vivo by chitosan hydrogels | Academy of Military Medical Sciences |
| Osteoarthritis & Cartilage | 2010 | Elevated osteopontin level of synovial fluid and articular cartilage is associated with disease severity in knee osteoarthritis patients | Central South University |
| Osteoarthritis & Cartilage | 2010 | Association of single-nucleotide polymorphisms in HLA class II/III region with knee osteoarthritis | Nanjing University/The University of Western Australia |
| Osteoarthritis & Cartilage | 2011 | IL-17RA aptamer-mediated repression of IL-6 inhibits synovium inflammation in a murine model of osteoarthritis | Wuhan University |
| Osteoarthritis & Cartilage | 2011 | MicroRNAs of rat articular cartilage at different developmental stages identified by Solexa sequencing | Xi'an Jiaotong University |
| Arthritis & Rheumatology | 2012 | Difference in Subchondral Cancellous Bone Between Postmenopausal Women With Hip Osteoarthritis and Osteoporotic Fracture Implication for Fatigue Microdamage, Bone Microarchitecture, and Biomechanical Properties | Shanghai Jiao Tong University |
| Arthritis & Rheumatology | 2012 | Genetic Inhibition of Fibroblast Growth Factor Receptor 1 in Knee Cartilage Attenuates the Degeneration of Articular Cartilage in Adult Mice | Army Medical University |
| Osteoarthritis & Cartilage | 2012 | Expression of microRNAs during chondrogenesis of human adipose-derived stem cells | Sun Yat-sen University |
| Osteoarthritis & Cartilage | 2012 | Enhanced COMP catabolism detected in serum of patients with arthritis and animal disease models through a novel capture ELISA | Shandong University/New York University |
| Osteoarthritis & Cartilage | 2012 | MicroRNA-337 is associated with chondrogenesis through regulating TGFBR2 expression | Xi'an Jiaotong University |
| Osteoarthritis & Cartilage | 2012 | Icariin: a potential promoting compound for cartilage tissue engineering | Sichuan University |
| Osteoarthritis & Cartilage | 2012 | Limb Idleness Index (LII): a novel measurement of pain in a rat model of osteoarthritis | The Chinese University of Hong Kong |
| Osteoarthritis & Cartilage | 2012 | Collagen fibril stiffening in osteoarthritic cartilage of human beings revealed by atomic force microscopy | The University of Hong Kong |
| Osteoarthritis & Cartilage | 2013 | Exogenous bFGF promotes articular cartilage repair via up-regulation of multiple growth factors | Peking Union Medical College Hospital |
| Osteoarthritis & Cartilage | 2013 | Monitoring wound healing of elastic cartilage using multiphoton microscopy | Fujian Normal University |
| Osteoarthritis & Cartilage | 2013 | Usefulness of specific OA biomarkers, thrombin-cleaved osteopontin, in the posterior cruciate ligament OA rabbit model | Central South University |
| Osteoarthritis & Cartilage | 2013 | Association between aspartic acid repeat polymorphism of the asporin gene and susceptibility to knee osteoarthritis: a genetic meta-analysis | Tianjin Hospital |
| Osteoarthritis & Cartilage | 2013 | Bone loss at subchondral plate in knee osteoarthritis patients with hypertension and type 2 diabetes mellitus | The University of Hong Kong |
| Osteoarthritis & Cartilage | 2013 | Spatial and temporal changes of subchondral bone proceed to microscopic articular cartilage degeneration in guinea pigs with spontaneous osteoarthritis | The University of Hong Kong |
| Osteoarthritis & Cartilage | 2013 | Basic science and clinical application of platelet-rich plasma for cartilage defects and osteoarthritis: a review | The General Hospital of Chinese People's Liberation Army |
| Osteoarthritis & Cartilage | 2018 | Bone morphogenetic proteins for articular cartilage regeneration | Central South University |
| Annals of the Rheumatic Diseases | 2014 | Evaluation of synovial angiogenesis in patients with rheumatoid arthritis using Ga-68-PRGD2 PET/CT: a prospective proof-of-concept cohort study | Peking Union Medical College Hospital |
| Annals of the Rheumatic Diseases | 2014 | Enhancement of the synthesis of n-3 PUFAs in fat-1 transgenic mice inhibits mTORC1 signalling and delays surgically induced osteoarthritis in comparison with wild-type mice | Southern Medical University |
| Annals of the Rheumatic Diseases | 2014 | ADAMTS-7 forms a positive feedback loop with TNF-alpha in the pathogenesis of osteoarthritis | Shandong University/New York University |
| Annals of the Rheumatic Diseases | 2014 | Cross-sectional and longitudinal associations between systemic, subchondral bone mineral density and knee cartilage thickness in older adults with or without radiographic osteoarthritis | Shanghai University of Traditional Chinese Medicine/University of Tasmania/Monash University |
| Arthritis & Rheumatology | 2014 | Long Noncoding RNA Related to Cartilage Injury Promotes Chondrocyte Extracellular Matrix Degradation in Osteoarthritis | Peking University |
| Arthritis & Rheumatology | 2014 | Early Response of Mouse Joint Tissue to Noninvasive Knee Injury Suggests Treatment Targets | Sun Yat-sen University |
| Arthritis & Rheumatology | 2014 | Identification of alpha 2-Macroglobulin as a Master Inhibitor of Cartilage-Degrading Factors That Attenuates the Progression of Posttraumatic Osteoarthritis | Shanxi Medical University |
| Arthritis & Rheumatology | 2014 | Distribution and Alteration of Lymphatic Vessels in Knee Joints of Normal and Osteoarthritic Mice | Shanghai University of Traditional Chinese Medicine |
| Arthritis & Rheumatology | 2014 | Chondrocyte beta-Catenin Signaling Regulates Postnatal Bone Remodeling Through Modulation of Osteoclast Formation in a Murine Model | Tianjin Medical University |
| Osteoarthritis & Cartilage | 2014 | Mass effect and signal intensity alteration in the suprapatellar fat pad: associations with knee symptoms and structure | Anhui Medical University/University of Tasmania/Monash University |
| Osteoarthritis & Cartilage | 2014 | Bone-cartilage interface crosstalk in osteoarthritis: potential pathways and future therapeutic strategies | The General Hospital of Chinese People's Liberation Army |
| Osteoarthritis & Cartilage | 2014 | Identical subchondral bone microarchitecture pattern with increased bone resorption in rheumatoid arthritis as compared to osteoarthritis | Guangdong General Hospital/The University of Western Australia |
| Osteoarthritis & Cartilage | 2014 | The role of small leucine-rich proteoglycans in osteoarthritis pathogenesis | Southern Medical University |
| Osteoarthritis & Cartilage | 2014 | Parathyroid hormone (1-34) prevents cartilage degradation and preserves subchondral bone micro-architecture in guinea pigs with spontaneous osteoarthritis | Hebei Medical University |
| Osteoarthritis & Cartilage | 2014 | Effectiveness of continuous and pulsed ultrasound for the management of knee osteoarthritis: a systematic review and network meta-analysis | Central South University |
| Osteoarthritis & Cartilage | 2014 | Defective autophagy in chondrocytes with Kashin-Beck disease but higher than osteoarthritis | Xi'an Jiaotong University |
| Osteoarthritis & Cartilage | 2014 | Exaggerated inflammatory environment decreases BMP-2/ACS-induced ectopic bone mass in a rat model: implications for clinical use of BMP-2 | Shanghai Jiao Tong University |
| Osteoarthritis & Cartilage | 2014 | Low magnitude high frequency vibration accelerated cartilage degeneration but improved epiphyseal bone formation in anterior cruciate ligament transect induced osteoarthritis rat model | The Chinese University of Hong Kong |
| Osteoarthritis & Cartilage | 2014 | Loss of Vhl in cartilage accelerated the progression of age-associated and surgically induced murine osteoarthritis | Army Medical University Army Medical University |
| Rheumatology | 2014 | Associations between vitamin D receptor gene polymorphisms and osteoarthritis: an updated meta-analysis | Anhui Medical University |
| Annals of the Rheumatic Diseases | 2015 | A longitudinal study of the association between infrapatellar fat pad maximal area and changes in knee symptoms and structure in older adults | Anhui Medical University |
| Annals of the Rheumatic Diseases | 2015 | Progranulin protects against osteoarthritis through interacting with TNF-alpha and beta-Catenin signalling | Shandong University/New York University |
| Annals of the Rheumatic Diseases | 2015 | Cross-sectional and longitudinal associations between circulating leptin and knee cartilage thickness in older adults | Shanghai University of Traditional Chinese Medicine/University of Tasmania/Monash University |
| Annals of the Rheumatic Diseases | 2015 | Inhibition of Rac1 activity by controlled release of NSC23766 from chitosan microspheres effectively ameliorates osteoarthritis development in vivo | Zhejiang University |
| Arthritis & Rheumatology | 2015 | Down-Regulation of Rac GTPase-Activating Protein OCRL1 Causes Aberrant Activation of Rac1 in Osteoarthritis Development | Zhejiang University |
| Osteoarthritis & Cartilage | 2015 | Association of patellar bone marrow lesions with knee pain, patellar cartilage defect and patellar cartilage volume loss in older adults: a cohort study | Anhui Medical University/University of Tasmania/Monash University |
| Osteoarthritis & Cartilage | 2015 | Metabolic triggered inflammation in osteoarthritis | Anhui Medical University/University of Tasmania/Monash University |
| Osteoarthritis & Cartilage | 2015 | SOX9 is a regulator of ADAMTSs-induced cartilage degeneration at the early stage of human osteoarthritis | The General Hospital of Chinese People's Liberation Army/Beijing Institute of Biotechnology |
| Osteoarthritis & Cartilage | 2015 | Knee osteoarthritis and all-cause mortality: the Wuchuan Osteoarthritis Study | Peking University |
| Osteoarthritis & Cartilage | 2015 | Expression profile of long noncoding RNAs in cartilage from knee osteoarthritis patients | Sun Yat-sen University |
| Osteoarthritis & Cartilage | 2015 | CCL3 serves as a potential plasma biomarker in knee degeneration (osteoarthritis) | Sun Yat-sen University |
| Osteoarthritis & Cartilage | 2015 | Asporin and osteoarthritis | Southern Medical University |
| Osteoarthritis & Cartilage | 2015 | Electrical stimulation for pain relief in knee osteoarthritis: systematic review and network meta-analysis | Central South University |
| Osteoarthritis & Cartilage | 2015 | NOD2 pathway via RIPK2 and TBK1 is involved in the aberrant catabolism induced by T-2 toxin in chondrocytes | Xi'an Jiaotong University |
| Osteoarthritis & Cartilage | 2015 | Autophagy protects chondrocytes from glucocorticoids-induced apoptosis via ROS/Akt/FOXO3 signaling | Shanghai Jiao Tong University |
| Osteoarthritis & Cartilage | 2015 | Effectiveness of low-level laser therapy in patients with knee osteoarthritis: a systematic review and meta-analysis | Sichuan University/Duke University |
| Osteoarthritis & Cartilage | 2015 | Joint distraction attenuates osteoarthritis by reducing secondary inflammation, cartilage degeneration and subchondral bone aberrant change | The Chinese University of Hong Kong |
| Osteoarthritis & Cartilage | 2015 | Bone turnover and articular cartilage differences localized to subchondral cysts in knees with advanced osteoarthritis | The University of Hong Kong |
| Osteoarthritis & Cartilage | 2015 | The emerging role of endothelin-1 in the pathogenesis of subchondral bone disturbance and osteoarthritis | The University of Hong Kong |
| Annals of the Rheumatic Diseases | 2016 | Signal intensity alteration in the infrapatellar fat pad at baseline for the prediction of knee symptoms and structure in older adults: a cohort study | Anhui Medical University/University of Tasmania/Monash University |
| Annals of the Rheumatic Diseases | 2016 | Halofuginone attenuates osteoarthritis by inhibition of TGF- activity and H-type vessel formation in subchondral bone | Southern Medical University/Johns Hopkins University |
| Annals of the Rheumatic Diseases | 2016 | Examination of overall treatment effect and the proportion attributable to contextual effect in osteoarthritis: meta-analysis of randomised controlled trials | Affiliated Hospital of University of Electronic Science and Technology |
| Arthritis & Rheumatology | 2016 | The Prevalence of Symptomatic Knee Osteoarthritis in China Results From the China Health and Retirement Longitudinal Study | Peking University |
| Arthritis & Rheumatology | 2016 | Fibroblast Growth Factor Receptor 3 Inhibits Osteoarthritis Progression in the Knee Joints of Adult Mice | Army Medical University Army Medical University |
| Osteoarthritis & Cartilage | 2016 | A novel method for assessing signal intensity within infrapatellar fat pad on MR images in patients with knee osteoarthritis | Anhui Medical University/University of Tasmania/University of Sydney |
| Osteoarthritis & Cartilage | 2016 | Vitamin D prevents articular cartilage erosion by regulating collagen II turnover through TGF-beta 1 in ovariectomized rats | Peking University |
| Osteoarthritis & Cartilage | 2016 | MicroRNA-320 regulates matrix metalloproteinase-13 expression in chondrogenesis and interleukin-1 beta-induced chondrocyte responses | Sun Yat-sen University |
| Osteoarthritis & Cartilage | 2016 | Parathyroid hormone(1-34) exhibits more comprehensive effects than celecoxib in cartilage metabolism and maintaining subchondral bone micro-architecture in meniscectomized guinea pigs | Hebei Medical University |
| Osteoarthritis & Cartilage | 2016 | MicroRNA221-3p modulates Ets-1 expression in synovial fibroblasts from patients with osteoarthritis of temporomandibular joint | Wuhan University |
| Osteoarthritis & Cartilage | 2016 | Establishment of a rabbit model to study the influence of advanced glycation end products accumulation on osteoarthritis and the protective effect of pioglitazone | Hunan Normal University |
| Osteoarthritis & Cartilage | 2016 | Hypoxia regulates sumoylation pathways in intervertebral disc cells: implications for hypoxic adaptations | Southeast University |
| Osteoarthritis & Cartilage | 2016 | Danshen prevents articular cartilage degeneration via antioxidation in rabbits with osteoarthritis | Xi'an Jiaotong University |
| Osteoarthritis & Cartilage | 2016 | Unilateral anterior crossbite induces aberrant mineral deposition in degenerative temporomandibular cartilage in rats | Air Force Medical University |
| Osteoarthritis & Cartilage | 2016 | Adipose-derived stem cells induce autophagic activation and inhibit catabolic response to pro-inflammatory cytokines in rat chondrocytes | Fudan University |
| Osteoarthritis & Cartilage | 2016 | Leptin promotes apoptosis and inhibits autophagy of chondrocytes through upregulating lysyl oxidase-like 3 during osteoarthritis pathogenesis | Zhejiang Chinese Medical University |
| Osteoarthritis & Cartilage | 2016 | Exogenous fibroblast growth factor 9 attenuates cartilage degradation and aggravates osteophyte formation in post-traumatic osteoarthritis | Army Medical University Army Medical University |
| Rheumatology | 2016 | Identification of IL-7 as a candidate disease mediator in osteoarthritis in Chinese Han population: a case-control study | Shanghai Jiao Tong University |
| Annals of the Rheumatic Diseases | 2017 | Kdm6b regulates cartilage development and homeostasis through anabolic metabolism | Zhejiang University |
| Osteoarthritis & Cartilage | 2017 | Cross-sectional and longitudinal associations between serum inflammatory cytokines and knee bone marrow lesions in patients with knee osteoarthritis | Anhui Medical University/University of Tasmania/Monash University |
| Osteoarthritis & Cartilage | 2017 | Associations between proximal tibiofibular joint (PTFJ) types and knee osteoarthritic changes in older adults | Anhui Medical University/University of Tasmania/Monash University |
| Osteoarthritis & Cartilage | 2017 | The effects of different doses of IGF-1 on cartilage and subchondral bone during the repair of full-thickness articular cartilage defects in rabbits | Peking Union Medical College Hospital |
| Osteoarthritis & Cartilage | 2017 | Regenerative approaches for cartilage repair in the treatment of osteoarthritis | Peking Union Medical College Hospital |
| Osteoarthritis & Cartilage | 2017 | MicroRNA-92a-3p regulates the expression of cartilage-specific genes by directly targeting histone deacetylase 2 in chondrogenesis and degradation | Sun Yat-sen University |
| Osteoarthritis & Cartilage | 2017 | mTORC1 activation downregulates FGFR3 and PTH/PTHrP receptor in articular chondrocytes to initiate osteoarthritis | Southern Medical University |
| Osteoarthritis & Cartilage | 2017 | Associations between serum ghrelin and knee symptoms, joint structures and cartilage or bone biomarkers in patients with knee osteoarthritis | Southern Medical University/University of Tasmania/Southern Medical University |
| Osteoarthritis & Cartilage | 2017 | Association between smoking and risk of knee osteoarthritis: a systematic review and meta-analysis | Hebei Medical University |
| Osteoarthritis & Cartilage | 2017 | Chondrogenic progenitor cells promote vascular endothelial growth factor expression through stromal-derived factor-1 | Harbin Medical University/University of Iowa |
| Osteoarthritis & Cartilage | 2017 | The rs4238326 polymorphism in ALDH1A2 gene potentially associated with non-post traumatic knee osteoarthritis susceptibility: a two-stage population-based study | Nantong University |
| Osteoarthritis & Cartilage | 2017 | A bioinformatic analysis of microRNAs role in osteoarthritis | China Medical University |
| Osteoarthritis & Cartilage | 2017 | Genome-wide DNA methylation profiling of articular cartilage reveals significant epigenetic alterations in Kashin-Beck disease and osteoarthritis | Xi'an Jiaotong University |
| Osteoarthritis & Cartilage | 2017 | The potential of induced pluripotent stem cells as a tool to study skeletal dysplasias and cartilage-related pathologic conditions | Xi'an Jiaotong University/ Umeå University |
| Osteoarthritis & Cartilage | 2017 | Altered expression of chondroitin sulfate structure modifying sulfotransferases in the articular cartilage from adult osteoarthritis and Kashin-Beck disease | Xi'an Jiaotong University |
| Osteoarthritis & Cartilage | 2017 | Matrix replenishing by BMSCs is beneficial for osteoarthritic temporomandibular joint cartilage | Air Force Medical University |
| Osteoarthritis & Cartilage | 2017 | Transcultural adaptation and validation of the Chinese version of the intermittent and constant osteoarthritis pain (ICOAP) measure in patients with knee osteoarthritis | Naval Medical University |
| Osteoarthritis & Cartilage | 2017 | Efficacy and safety of duloxetine in Chinese patients with chronic pain due to osteoarthritis: a randomized, double- blind, placebo-controlled study | Lilly Suzhou Pharmaceutical Co. Ltd |
| Osteoarthritis & Cartilage | 2017 | Lentiviral vector-mediated shRNAs targeting a functional isoform of the leptin receptor (Ob-Rb) inhibit cartilage degeneration in a rat model of osteoarthritis | Sichuan University |
| Osteoarthritis & Cartilage | 2017 | Intra-articular injection of microRNA-140 (miRNA-140) alleviates osteoarthritis (OA) progression by modulating extracellular matrix (ECM) homeostasis in rats | Sichuan University |
| Osteoarthritis & Cartilage | 2017 | Stepwise preconditioning enhances mesenchymal stem cell-based cartilage regeneration through epigenetic modification | The Chinese University of Hong Kong |
| Osteoarthritis & Cartilage | 2017 | Attenuation of subchondral bone abnormal changes in osteoarthritis by inhibition of SDF-1 signaling | The Chinese University of Hong Kong/Bao-An District People's Hospital |
| Osteoarthritis & Cartilage | 2017 | Cartilage-specific deletion of Alk5 gene results in a progressive osteoarthritis-like phenotype in mice | Army Medical University |
| Annals of the Rheumatic Diseases | 2018 | Synovial macrophage M1 polarisation exacerbates experimental osteoarthritis partially through R-spondin-2 | Southern Medical University |
| Annals of the Rheumatic Diseases | 2018 | Tyrosine kinase Fyn promotes osteoarthritis by activating the -catenin pathway | Southern Medical University |
| Arthritis & Rheumatology | 2018 | Loading-Induced Reduction in Sclerostin as a Mechanism of Subchondral Bone PlateSclerosis in Mouse Knee Joints During Late-Stage Osteoarthritis | Shihezi University/Shandong University/University of Pennsylvania |
| Osteoarthritis & Cartilage | 2018 | Signal intensity alteration within infrapatellar fat pad predicts knee replacement within 5 years: data from the Osteoarthritis Initiative | Anhui Medical University/University of Tasmania/University of Sydney/Southern Medical University |
| Osteoarthritis & Cartilage | 2018 | Human umbilical cord Wharton's jelly mesenchymal stem cells combined with an acellular cartilage extracellular matrix scaffold improve cartilage repair compared with microfracture in a caprine model | The General Hospital of Chinese People's Liberation Army |
| Osteoarthritis & Cartilage | 2018 | The burden for knee osteoarthritis among Chinese elderly: estimates from a nationally representative study | Peking University |
| Osteoarthritis & Cartilage | 2018 | Intra-articular injection of mesenchymal stem cells in treating knee osteoarthritis: a systematic review of animal studies | Peking University/Lanzhou University |
| Osteoarthritis & Cartilage | 2018 | The importance of synovial inflammation in osteoarthritis: current evidence from imaging assessments and clinical trials | Southern Medical University/University of Tasmania |
| Osteoarthritis & Cartilage | 2018 | Systemic and local adipose tissue in knee osteoarthritis | Southern Medical University/University of Tasmania |
| Osteoarthritis & Cartilage | 2018 | A novel method for assessing proximal tibiofibular joint on MR images in patients with knee osteoarthritis | Southern Medical University/University of Tasmania/Anhui Medical University |
| Osteoarthritis & Cartilage | 2018 | Associations between knee structural measures, circulating inflammatory factors and MMP13 in patients with knee osteoarthritis | Anhui Medical University/Southern Medical University/University of Tasmania |
| Osteoarthritis & Cartilage | 2018 | Shape-memory collagen scaffold for enhanced cartilage regeneration: native collagen versus denatured collagen | Fudan University |
| Osteoarthritis & Cartilage | 2018 | Knocking out or pharmaceutical inhibition of fatty acid binding protein 4 (FABP4) alleviates osteoarthritis induced by high-fat diet in mice | The University of Hong Kong |
| Osteoarthritis & Cartilage | 2018 | Immediate and short-term effects of gait retraining on the knee joint moments and symptoms in patients with early tibiofemoral joint osteoarthritis: a randomized controlled trial | The Hong Kong Polytechnic University |
| Osteoarthritis & Cartilage | 2018 | Biphasic hierarchical extracellular matrix scaffold for osteochondral defect regeneration | Zhejiang University |
| Osteoarthritis & Cartilage | 2018 | A novel FGFR1-binding peptide attenuates the degeneration of articular cartilage in adult mice | Army Medical University Army Medical University |
| Rheumatology | 2018 | Current status and future prospects for disease modification in osteoarthritis | Southern Medical University/Guangdong Second Provincial General Hospital/University of Tasmania |
| Rheumatology | 2018 | Comparative advantages of infrapatellar fat pad: an emerging stem cell source for regenerative medicine | Subei People's Hospital/West Virginia University |
| Annals of the Rheumatic Diseases | 2019 | Single-cell RNA-seq analysis reveals the progression of human osteoarthritis | The General Hospital of Chinese People's Liberation Army/Peking University |
| Annals of the Rheumatic Diseases | 2019 | PPAR gamma preservation via promoter demethylation alleviates osteoarthritis in mice | Nanjing University |
| Annals of the Rheumatic Diseases | 2019 | Wnt16 attenuates osteoarthritis progression through a PCP/JNK-mTORC1-PTHrP cascade | The Chinese University of Hong Kong |
| Annals of the Rheumatic Diseases | 2019 | CircSERPINE2 protects against osteoarthritis by targeting miR-1271 and ETS-related gene | Zhejiang University |
| Arthritis & Rheumatology | 2019 | Attenuated Joint Tissue Damage Associated With Improved Synovial Lymphatic Function Following Treatment With Bortezomib in a Mouse Model of Experimental Posttraumatic Osteoarthritis | Henan Normal University/University of Rochester Medical Center |
| Osteoarthritis & Cartilage | 2019 | Associations between serum S100A8/S100A9 and knee symptoms, joint structures and cartilage enzymes in patients with knee osteoarthritis | Anhui Medical University/Southern Medical University/University of Tasmania |
| Osteoarthritis & Cartilage | 2019 | Ambulatory activity interacts with common risk factors for osteoarthritis to modify increases in MRI-detected osteophytes | Southern Medical University/University of Tasmania/Monash University/Anhui Medical University |
| Osteoarthritis & Cartilage | 2019 | Risk factor heterogeneity for medial and lateral compartment knee osteoarthritis: analysis of two prospective cohorts | Central South University/Harvard University |
| Osteoarthritis & Cartilage | 2019 | Thiazide diuretics and risk of knee replacement surgery among patients with knee osteoarthritis: a general population-based cohort study | Central South University/Harvard University |
| Osteoarthritis & Cartilage | 2019 | Intra-articular corticosteroids and the risk of knee osteoarthritis progression: results from the Osteoarthritis Initiative | Central South University/Harvard University |
| Osteoarthritis & Cartilage | 2019 | Gut microbiota and obesity-associated osteoarthritis | Shanghai University of Sport |
| Osteoarthritis & Cartilage | 2019 | Cartilage oligomeric matrix protein, C-terminal cross-linking telopeptide of type II collagen, and matrix metalloproteinase-3 as biomarkers for knee and hip osteoarthritis (OA) diagnosis: a systematic review and meta-analysis | Shanxi Medical University/Shanxi Medical Health Media Group Co.,Ltd |
| Osteoarthritis & Cartilage | 2019 | Maintenance of SOX9 stability and ECM homeostasis by selenium-sensitive PRMT5 in cartilage | Xi'an Jiaotong University |
| Osteoarthritis & Cartilage | 2019 | Clock mutant promotes osteoarthritis by inhibiting the acetylation of NF kappa B | Fudan University |
| Osteoarthritis & Cartilage | 2019 | Intra-articular injection of magnesium chloride attenuates osteoarthritis progression in rats | The Chinese University of Hong Kong |
| Osteoarthritis & Cartilage | 2019 | Risk factors for falls in patients with total hip arthroplasty and total knee arthroplasty: a systematic review and meta-analysis | The Hong Kong Polytechnic University |
| Osteoarthritis & Cartilage | 2019 | NGF increases FGF2 expression and promotes endothelial cell migration and tube formation through PI3K/Akt and ERK/MAPK pathways in human chondrocytes | Zhejiang University |
| Rheumatology | 2019 | MicroRNA-21-5p as a novel therapeutic target for osteoarthritis | Xuzhou Medical University |

The calculation standards: (1) If the corresponding author (including co-corresponding) or the first author (including co-first) came from Chinese institutions, the paper was included in this table. (2) The affiliations of corresponding author (including co-corresponding author) were listed out in the table. If there are N corresponding authors in one paper, each author counts 1/N when calculated. If the affiliations of corresponding author (including co-corresponding) were all foreign institutions, the affiliations of the first author (including co-first author) were recorded. (3) If an author has multiple institutions, the first Chinese affiliation were recorded and calculated.
