# Supplementary figures and images for: Progress in osteoarthritis research by the National Natural Science Foundation of China
Source: Bone Res. 2022 May 24;10:41. doi: 10.1038/s41413-022-00207-y (PMC9130253; doi:10.1038/s41413-022-00207-y)

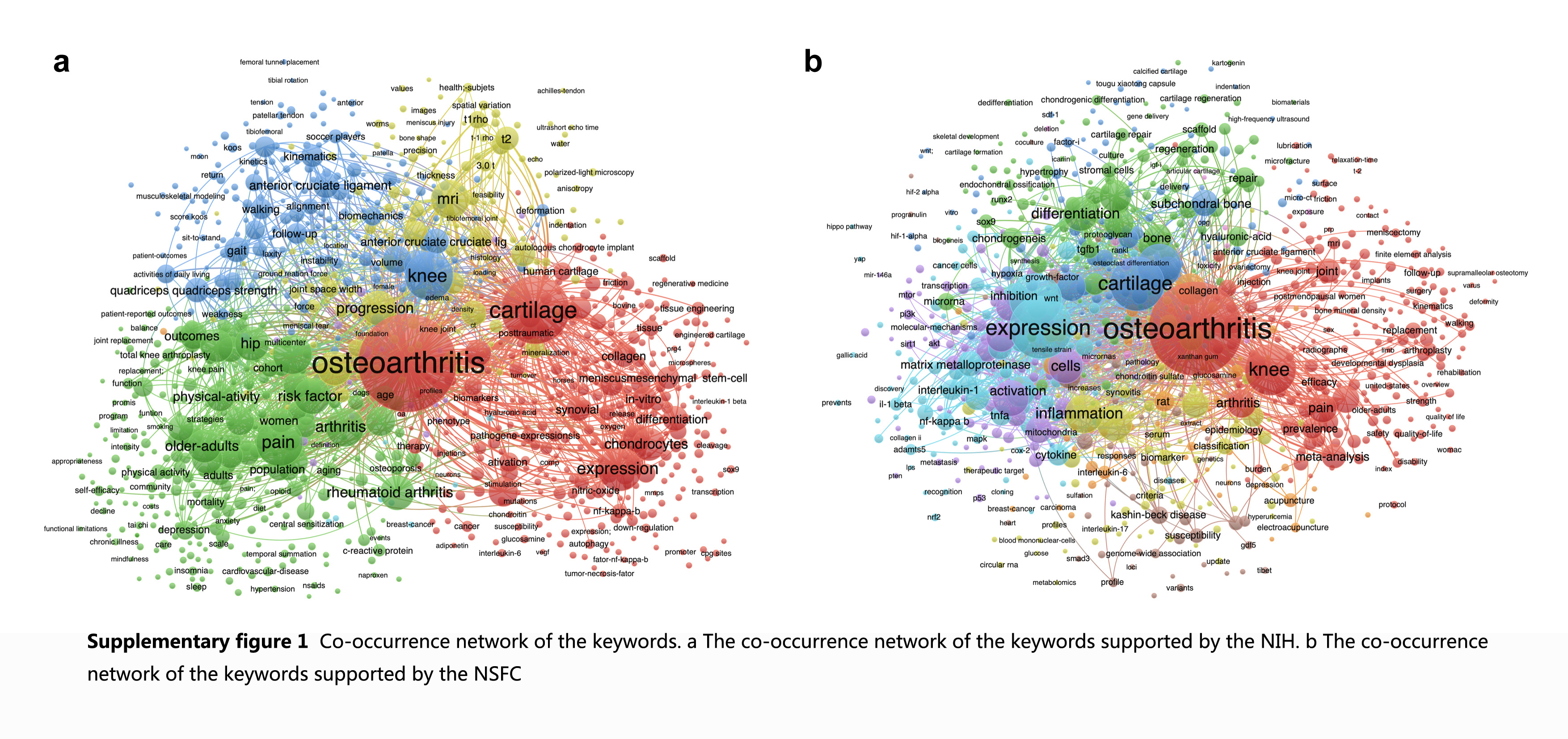

Supplement: Supplementary file 4 — Figure 1 [file 41413_2022_207_MOESM4_ESM.jpg]
